# Development of arthroscopy: contributions of Masaki Watanabe and Japan

**DOI:** 10.1007/s00264-026-06948-3

**Published:** 2026-07-23

**Authors:** Shuji Taketomi, Sakae Tanaka

**Affiliations:** Department of Orthopaedic Surgery, Faculty of Medicine, The University of Tokyo, Tokyo, Japan

**Keywords:** Arthroscopes, Arthroscopy, Japan, Masaki Watanabe

## Abstract

The history of arthroscopy in Japan occupies a pivotal position in the global evolution of modern orthopaedic surgery. Its foundations were established by Kenji Takagi, who in 1918 became the first to visualize the knee joint cavity of a cadaver using a cystoscope. He subsequently developed a series of original arthroscopes, including models designed for clinical use and animal experimentation. His work demonstrated the feasibility of arthroscopy as a practical method for direct intra-articular visualization. This foundation was further advanced by Masaki Watanabe, who transformed arthroscopy from an experimental technique into a clinically applicable discipline. Watanabe developed improved arthroscopes suitable for routine practice, published the Atlas of Arthroscopy, and in 1962 performed the world’s first arthroscopic partial meniscectomy. His subsequent innovations, including the Model-21 arthroscope and later refinements in arthroscopic innovations, established the technical foundation of modern arthroscopic surgery. Through the efforts of Takagi and Watanabe, Japan not only led the technical advancement of arthroscopy but also played a decisive role in its global dissemination. Consequently, the history of arthroscopy in Japan represents a landmark chapter in the worldwide development of minimally invasive orthopaedic surgery.

## Introduction

The development of arthroscopy has had a profound impact on modern orthopaedic surgery and many be regarded as one of the most transformative advances in the history of orthopaedics. Many orthopedic procedures routinely performed today would not be possible without arthroscopy and spinal endoscopy. What, then, has been the significance of arthroscopy in the evolution of orthopaedic surgery?

First, arthroscopy has substantially enhanced our understanding of intra-articular pathology. Before its introduction, meniscal tears, anterior cruciate ligament (ACL) injuries, articular cartilage lesions, synovial disorders, and intra-articular loose bodies could only be inferred from clinical symptoms, physical examination findings, radiographic studies, or the limited observations available during open surgery. The advent of arthroscopy enabled direct in vivo visualization of these lesions, leading to rapid advances in pathological classification, understanding of injury morphology, and assessment of disease progression.

Second, arthroscopy established the foundation of minimally invasive surgery. Compared with conventional open procedures, arthroscopic surgery requires smaller incisions, minimizes soft tissue disruption, and reduces postoperative pain, hospitalization, infection risk, and joint stiffness. Although initially developed for the knee, arthroscopic techniques were subsequently applied to other joints, including the shoulder, ankle, elbow, hip, and wrist, as well as the spine. Consequently, patients were able to return to daily activities and sports more rapidly, laying the groundwork for modern sports orthopaedics.

Furthermore, arthroscopy fundamentally changed treatment strategies. A representative example is the management of meniscal injuries. Historically, arthroscopy enabled detailed evaluation of tear location and morphology, facilitating meniscal repair and promoting the shift from resection to preservation. A similar transformation occurred in the treatment of ACL injuries. The development of anatomical ACL reconstruction would not have been possible without arthroscopic techniques. Precise anatomical tunnel placement [1–3], double-bundle reconstruction [4, 5], rectangular tunnel reconstruction [6–8], and the simultaneous evaluation and treatment of meniscal ramp lesions [9] and root tears [10] have all been made possible through arthroscopy.

Arthroscopy has also contributed significantly to the standardization of diagnostic accuracy. Although magnetic resonance imaging (MRI) now provides excellent evaluation of intra-articular structures, arthroscopy allows direct visualization and precise assessment of these structures. In addition to evaluating morphology, surgeons can assess tissue quality and stability using a probe. Thus, the unique advantage of arthroscopy lies not only in visualization but also in its ability to facilitate direct intra-articular inspection and palpation.

Its influence on education has been equally substantial. Because arthroscopic images can be viewed simultaneously by the surgeon, assistants, and trainees, the teaching of intra-articular anatomy, pathological interpretation, and surgical techniques has become considerably more effective. The ability to record procedures has further facilitated surgical review, research, and the international dissemination of technical knowledge. In this regard, arthroscopy serves not only as a surgical instrument but also as a platform that has advanced education, research, and standardization within orthopaedic surgery.

Arthroscopy is one of the medical technologies of which Japan has earned international recognition, and the country has played pivotal role in the global development of arthroscopic surgery. This section reviews the history of arthroscopy in Japan, with particular emphasis on its evolution and contributions within the country.

##   The history of endoscope before the development of arthroscope

The origins of the arthroscope can be traced to the development of the endoscope. The history of endoscopy began in 1806, when Philipp Bozzini, a physician in the German army, introduced the “Lichtleiter,” a device that used a candle as its light source. He was the first person to insert a tube into the human body and observe the interior of a body cavity using illumination from an external source. Using the “Lichtleiter,” which consisted of two tubes, he attempted to examine the interior of the bladder [11]. Despite its innovative design, the instrument did not put achieve widespread practical use for more than half a century.

In 1853, the French urologist Antonin Jean Desormeaux used a direct-viewing cystoscope to examine the bladder and other internal structures [12]. He was the first to use the term *endoscope*, meaning an instrument for viewing the interior of a body cavity, and is therefore widely recognized as the father of endoscopy. As a light source, he burned a mixture of alcohol and turpentine oil and directed the reflected light through the scope for illumination. Before the invention of the incandescent bulb by Edison, the development of endoscopy was largely driven by innovations in light sources, including candles, gas lamps, and turpentine oil.

In 1876, the German physician Max Nitze introduced the “Kystoskope” [13]. This instrument enabled visualization of the bladder using a heated platinum loop that was cooled by circulating ice water. A decade later, in 1886, Nitze developed the first cystoscope equipped with an incandescent bulb [12], representing a major advance in endoscopic technology.

In 1910, Hans Christian Jacobaeus expanded the application of endoscopy by developing abdominal endoscopy and laparothoracoscopy and performing the first laparoscopic procedure [14, 15]. Through these advancements, endoscopy evolved into a technique capable of examining numerous tubular and hollow organs, as well as various body cavities. However, despite these achievements, access to the interior of synovial joints remained beyond the reach of endoscopic technology at that time.

## History of arthroscopy development in the world

In Europe and the United States, Eugen Bircher of Switzerland reported the results of arthroscopic examinations of 18 knees using a thoraco-laparoscope in 1921 [16]. Subsequently, in 1925, the American surgeon Phillip H. Kreuscher published the first paper dedicated to arthroscopy, entitled “Semilunar Cartilage Disease—A Plea for Early Recognition by Means of the Arthroscope” [12]. However, Kreuscher did not publish any further studies on the subject.

Additional investigation into arthroscopy were conducted by H. Finkelstein and L. Mayer, Michael S. Burman (1931), R. Sommer (1937), E. Vaubel (1938), and K. H. Wilcke (1939), among others. Nevertheless, most of these research efforts were discontinued after relatively short periods [17].

The development of arthroscopy faced several challenges. The narrow joint space, complex intra-articular anatomy, and the presence of osteochondral structures that restricted endoscope movement made joint visualization technically demanding. Furthermore, the technical difficulty of manufacturing sufficiently thin optical instruments likely contributed to the slow progress of arthroscopic technology during this period [17].

## History of arthroscopy in Japan

### Takagi’s arthroscope

Arthroscopy is one of the medical technologies for which Japan has earned international recognition. Since 1918, when Kenji Takagi, an orthopaedic surgeon at Tokyo Imperial University, first visualized the knee joint cavity of a cadaver using a cystoscope, Japan has played a leading role in the development of arthroscopy through continuous refinement and experimentation, ultimately contributing to the widespread use of the technique today.

Takagi modified the tip of a cystoscope and, in 1920, created a prototype that became the world’s first arthroscope. This instrument had a relatively large diameter of 7.3 mm and required an outer sheath, making it difficult to use in clinical practice. Nevertheless, Takagi successfully observed the inside of a joint by inserting the device through a fistula in a patient with tuberculous arthritis of the knee while distending the joint with normal saline. Subsequently, in collaboration with optical instrument manufacturers, Takagi repeatedly refined the device and developed a thinner arthroscope. In 1931, he succeeded in producing Takagi’s Model-1 arthroscope with a diameter of 3.5 mm [17].

Takagi remained highly committed to advancing arthroscopy and ultimately developed 12 arthroscope models (Takagi Models 1 through 12), including a 2.6-mm version designed for animal experiments, along with the necessary accessory instruments. He also investigated arthroscopic techniques for the knee, shoulder, elbow, hip, and ankle joints [17].

**Fig. 1:**
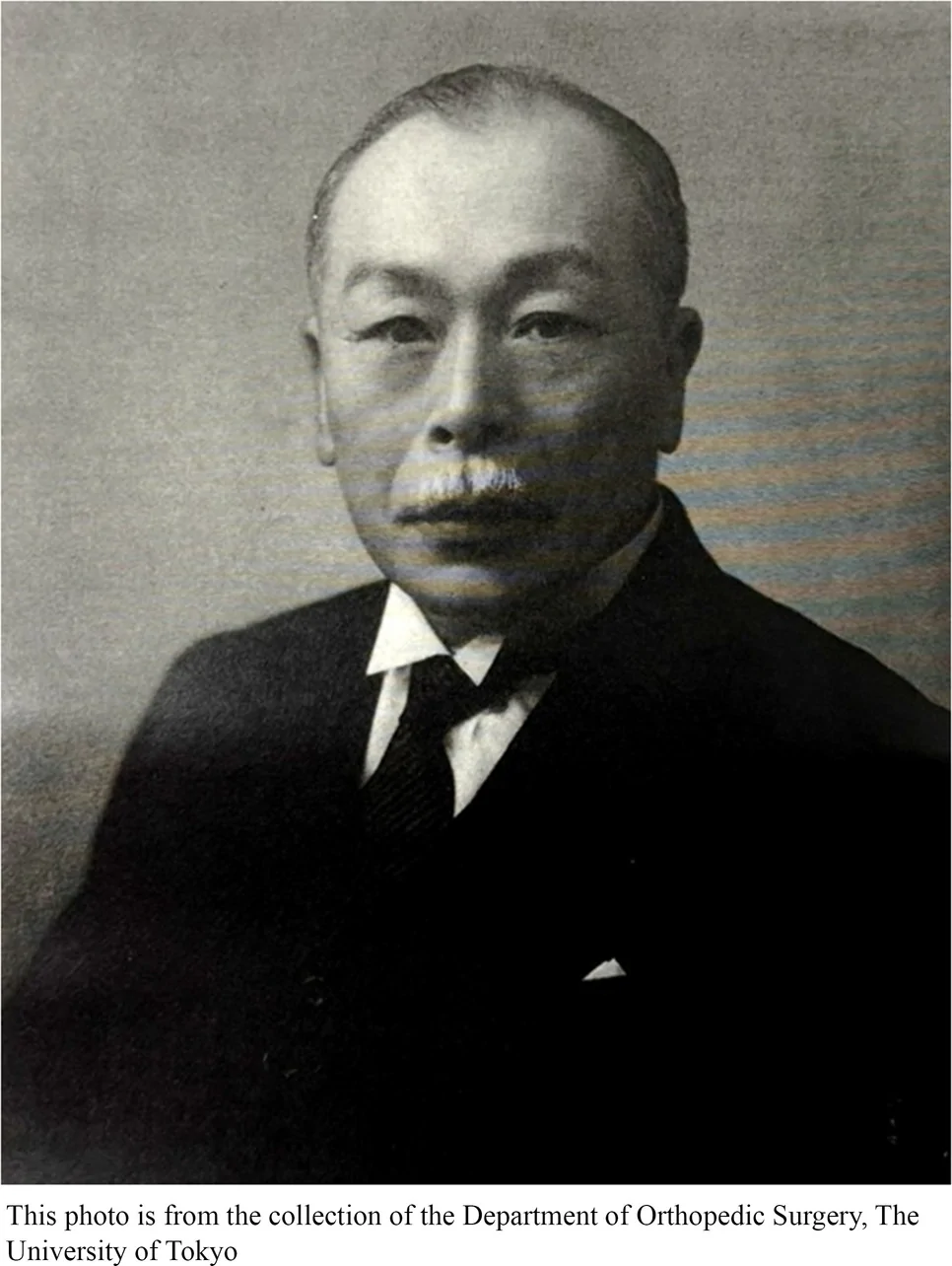
Kenji Takagi (1889–1963) This photo is from the collection of the Department of Orthopedic Surgery, The University of Tokyo

The advancement of arthroscopy during this period is attributed in part to Takagi’s strong engineering knowledge and his collaboration with the skilled craftsman Masaru Takei. Among Takagi’s 12 models, the Model-4 arthroscope featured an adjustable focus mechanism achieved by advancing and retracting the basal prism, allowing lateral viewing and producing a clear magnified image of a minimum working distance of < 3 mm. This instrument was exhibited at the 1937 Paris International Exposition, where it was reportedly “praised as the most exquisite” [17]. (Fig. 1)

### The father of arthroscopy: Masaki Watanabe

Masaki Watanabe inherited Kenji Takagi’s vision of establishing arthroscopy as a practical clinical tool. In 1937, Watanabe joined the Department of Orthopaedic Surgery at Tokyo Imperial University, where Takagi-centered arthroscopic research was gaining momentum. Under Takagi’s instruction to “perform arthroscopy of the horse ankle joint,” Watanabe used Takagi’s Model-12 arthroscope to conduct arthroscopic examinations of the ankle joints of military horses.

Watanabe was later transferred to Tokyo Teishin Hospital, where he continued his arthroscopic studies using the Model-12 arthroscope. During this period, he observed that arthroscopic procedures often alleviated knee pain in patients and actively applied adjunctive techniques such as joint lavage therapy, so-called joint pumping. However, the Model-12 arthroscope had a critical limitation in that it was fragile. Around 1950, when Watanabe needed to examine a female patient with a knee joint disorder, the Model-12 arthroscope was damaged and unusable. With no alternative available, he substituted a pediatric cystoscope and found that it allowed clear visualization of the intra-articular space.

This experience became the turning point for the development of a new arthroscope based on the pediatric cystoscope, leading to Watanabe’s Model-13 arthroscope [17]. The Model-13 had a 4-mm diameter, incorporated a light bulb positioned beside the lens, and was designed for oblique forward viewing from a posterior approach [17]. To facilitate routine clinical use, a standardized version was also commercialized and gradually adopted in clinical practice.

In 1957, based on approximately 300 arthroscopic examinations performed with the Model-13 arthroscope, Watanabe, together with Sakae Takeda and Hiroshi Ikeuchi, published Atlas of Arthroscopy [18]. The cases were organized by disease category and illustrated using watercolor drawings by the artist Shinichiro Fujihashi [17].

**Fig. 2:**
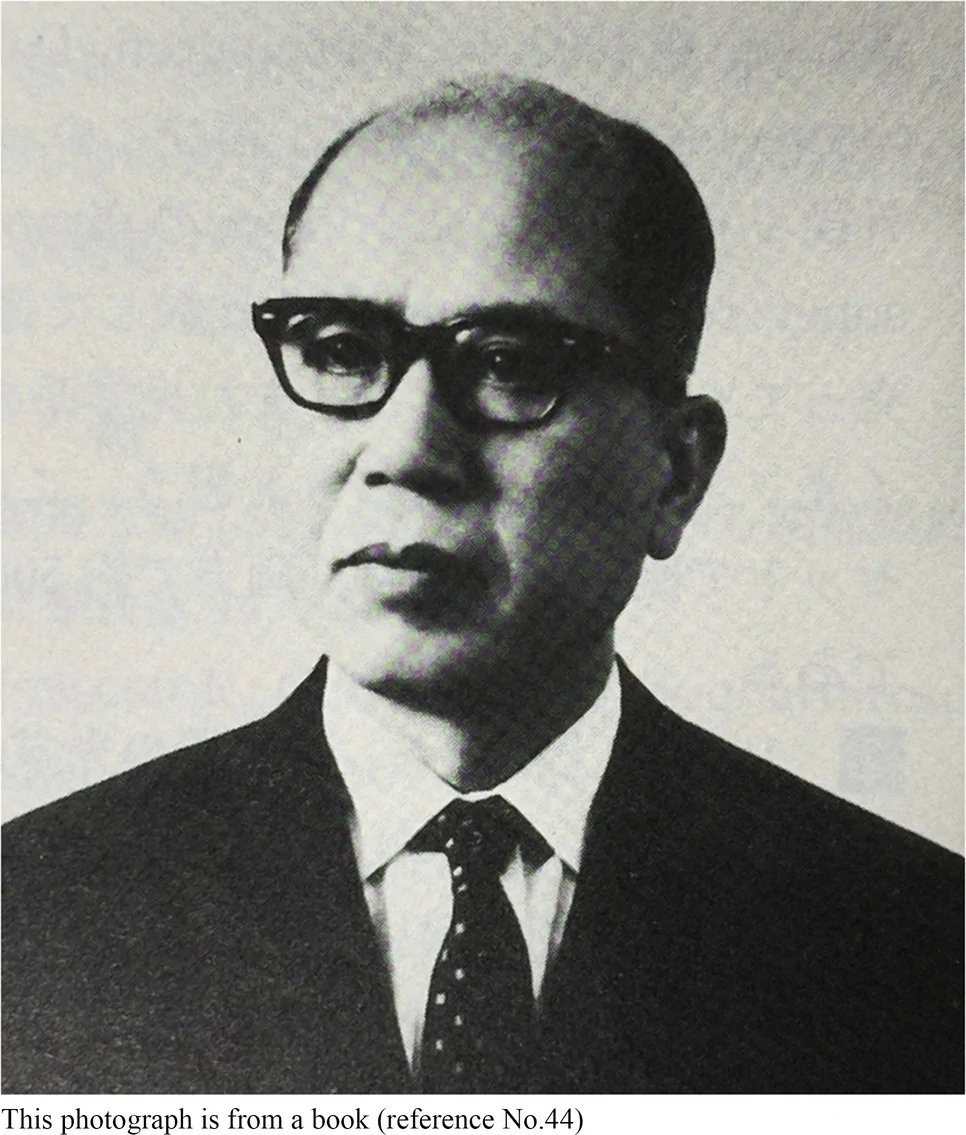
Masaki Watanabe(1911–1994) [19]

**Fig. 3:**
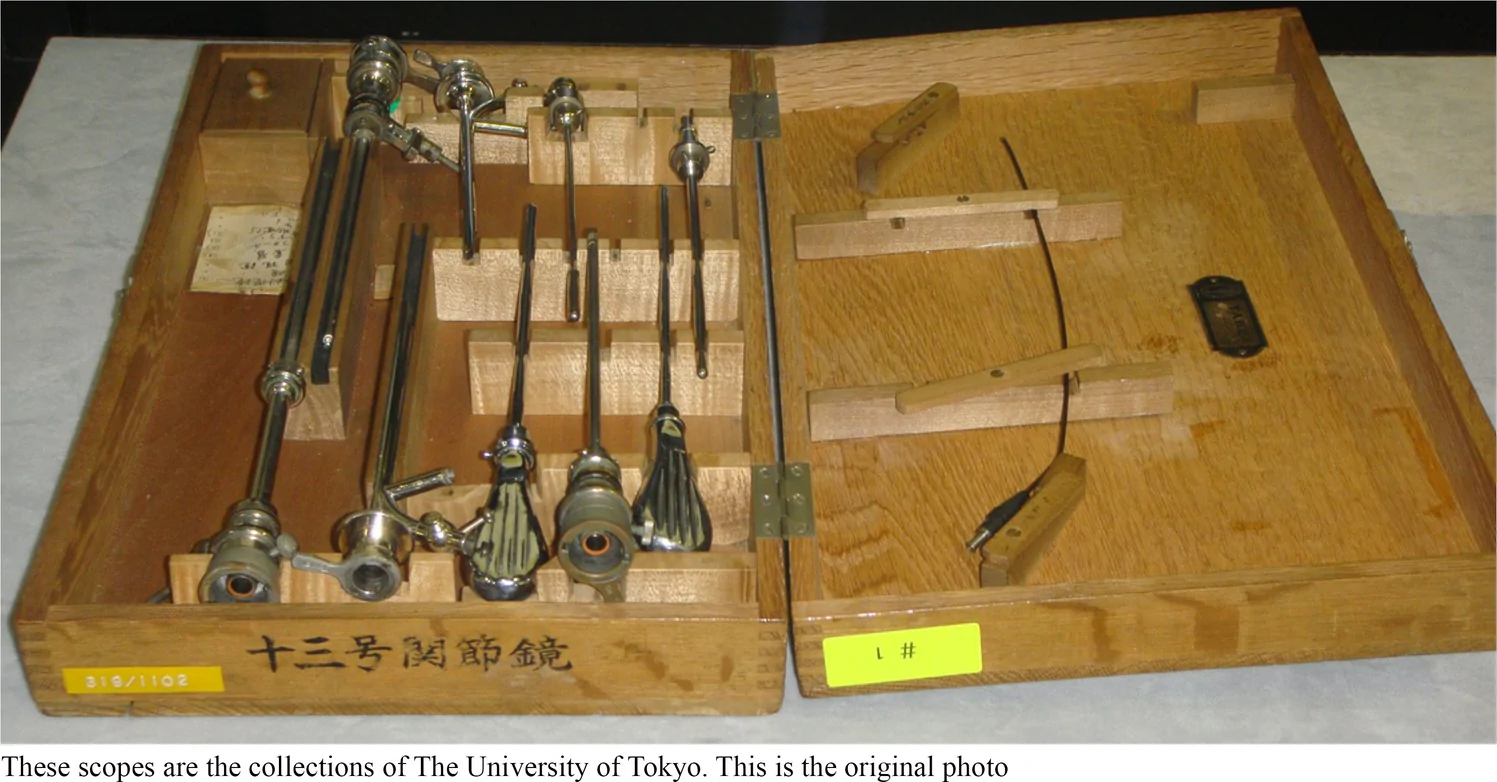
Watanabe’s Model-13 arthroscope

**Fig. 4:**
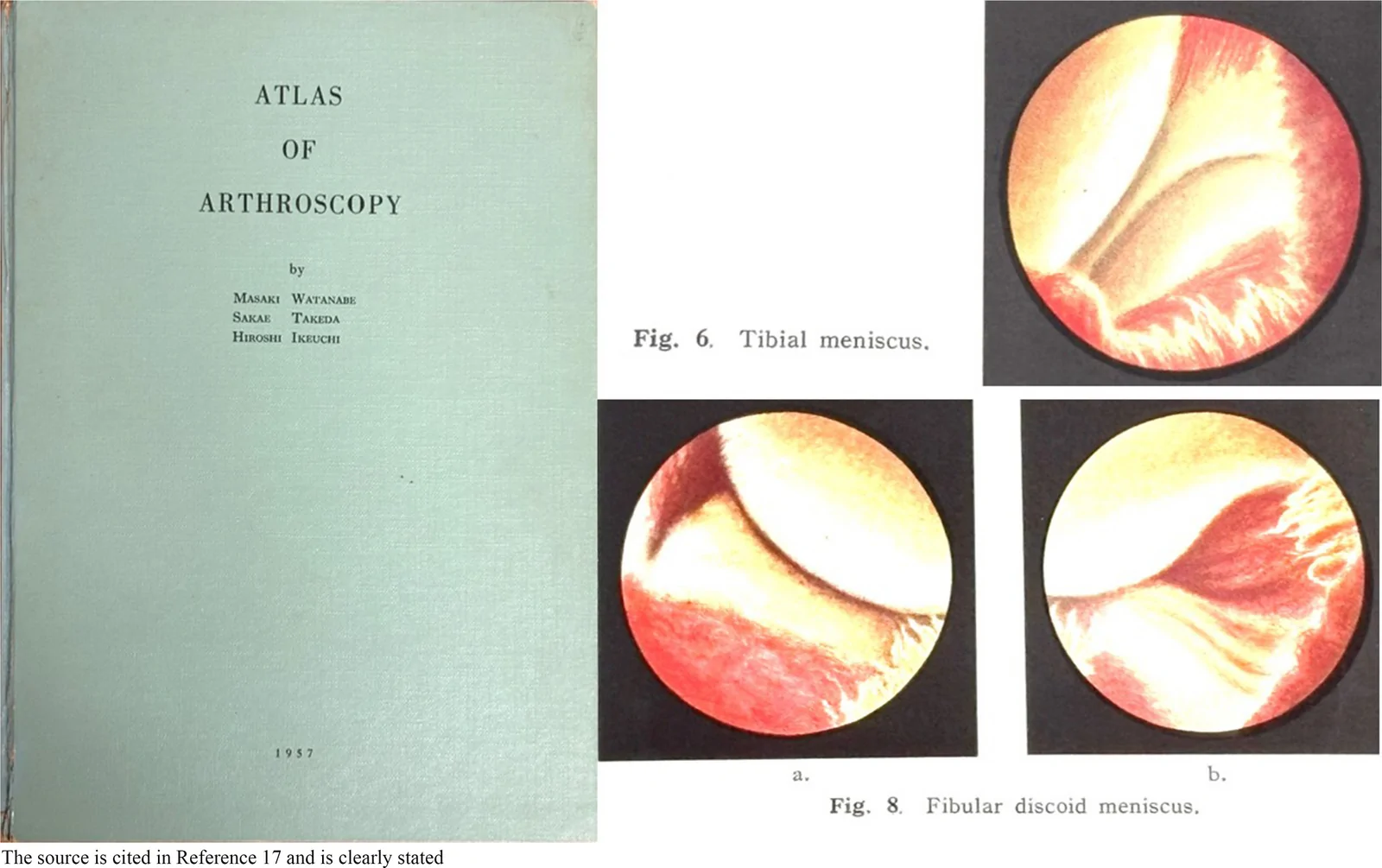
Atlas of Arthroscopy was published by Watanabe, Sakae Takeda, and Hiroshi Ikeuchi in 1957 [18]

The Model-14 was subsequently developed as a direct-view instrument with an increased lens diameter of 5.7 mm, thicker than the Model-13, to maximize brightness and improve image quality for color photography. At the 7th SICOT Congress held in Barcelona in 1957, a 16-mm color film recorded using with the Model-14 was presented. Thereafter, successive improvements were made from the Model-14 through the Model-20 series, with incremental refinements in design and performance [17] (Figs. 2, 3 and 4).

### The first arthroscopic partial meniscectomy in the world

Until the development of Watanabe’s Model-21 arthroscope, arthroscopy was primarily used as a diagnostic tool. Arthroscopic interventions were largely limited to synovial biopsy, removal of loose bodies, tumour excision, and synovectomy using a punch instrument. However, in 1962, Watanabe successfully performed the world’s first arthroscopic partial meniscectomy using the Model-21 arthroscope.

The patient was a 17-year-old basketball player diagnosed with a flap tear of the medial meniscus in the right knee. The torn fragment was arthroscopically resected and removed using scissors and forceps. The patient was able to walk home after the procedure and returned to basketball activity six weeks later [17].

In 1968, Hiroshi Ikeuchi achieved another milestone by performing the first arthroscopic total resection of a complete discoid lateral meniscus [20]. Over an eight year period, Watanabe, Takeda, and Ikeuchi collectively performed approximately 800 arthroscopic knee examinations. Building on these clinical experiences, they published the second edition of Atlas of Arthroscopy in 1969 [21].

In the same year, during the 11th SICOT Congress held in Mexico, they presented motion picture footage obtained with the Model-21 arthroscope. This presentation significantly increased international awareness of arthroscopy, particularly in Europe and the United States. Consequently, many orthopaedic surgeons, including R. W. Jackson of Canada and R. L. O’Connor of the United States, traveled to Japan to study arthroscopic techniques, and Watanabe began conducting instructional seminars. From 1970 onward, arthroscopic surgery rapidly expanded worldwide.

**Fig. 5:**
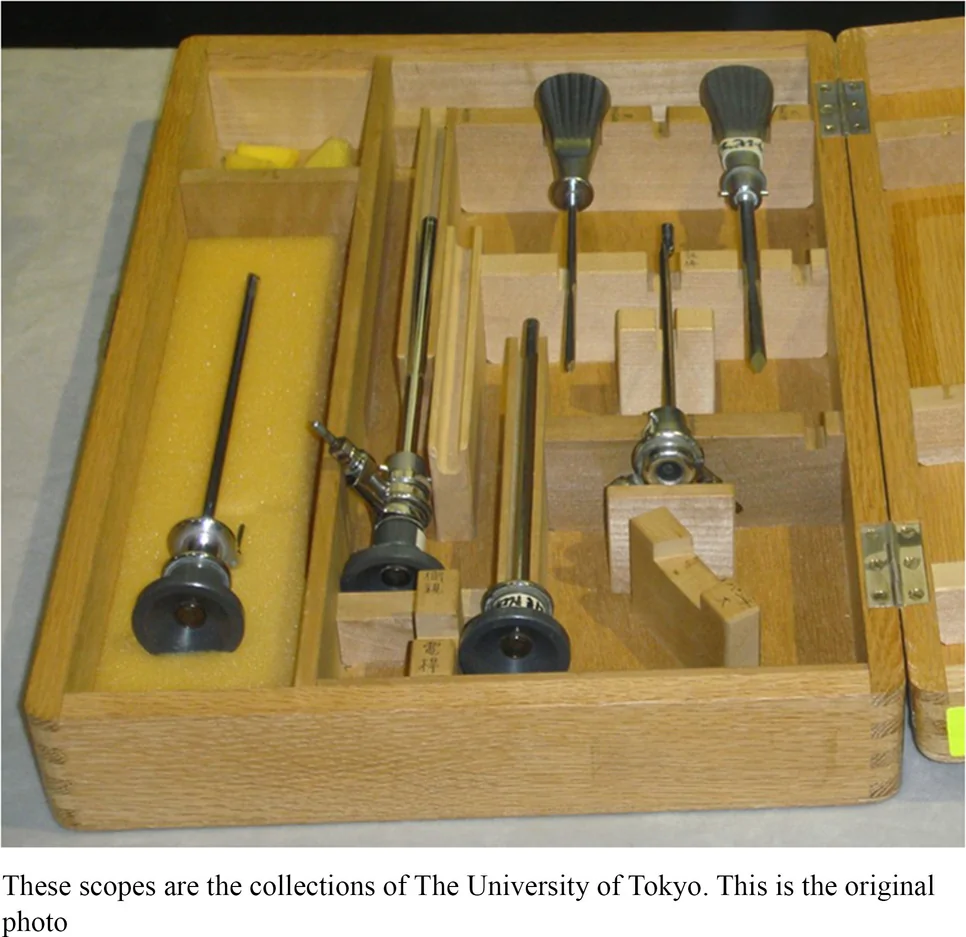
Watanabe’s Model-21 arthroscope

**Fig. 6:**
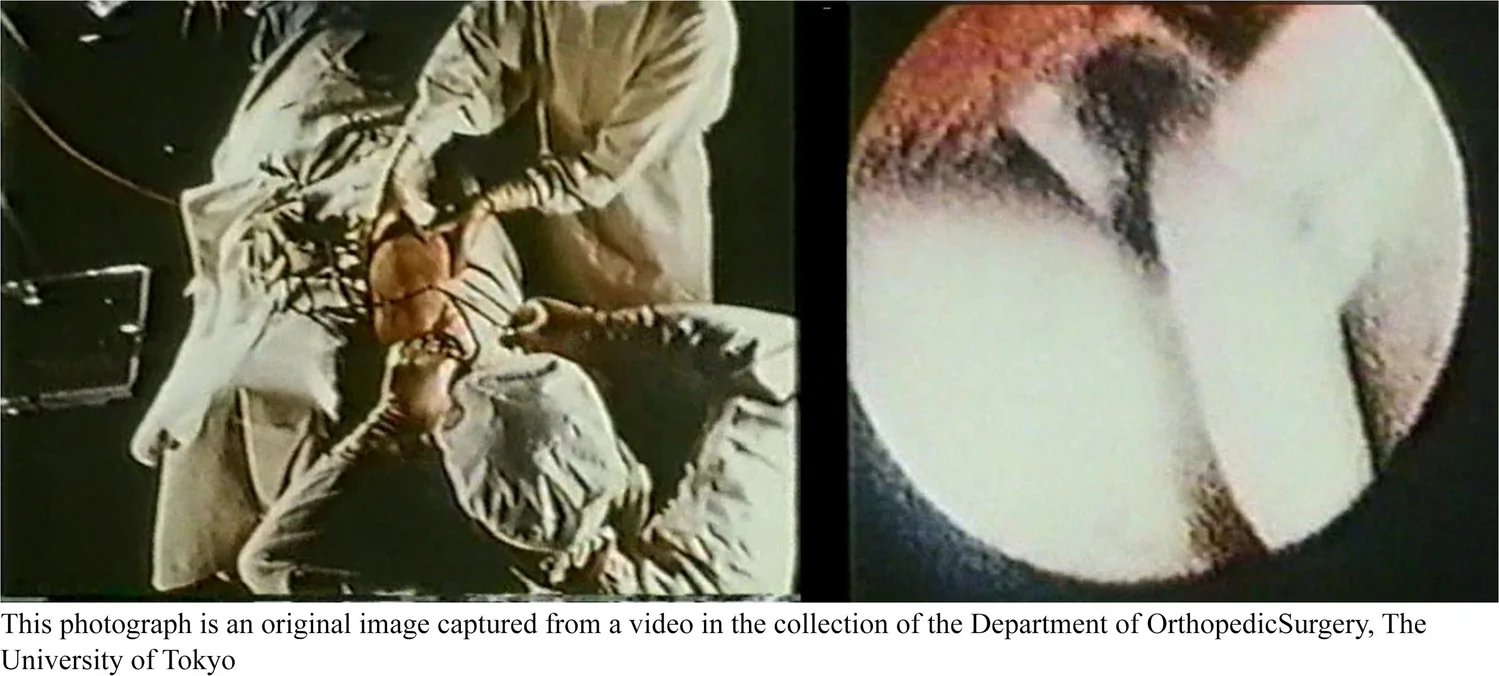
Movie of the world’s first arthroscopic partial meniscectomy using the Model-21 arthroscope

**Fig. 7:**
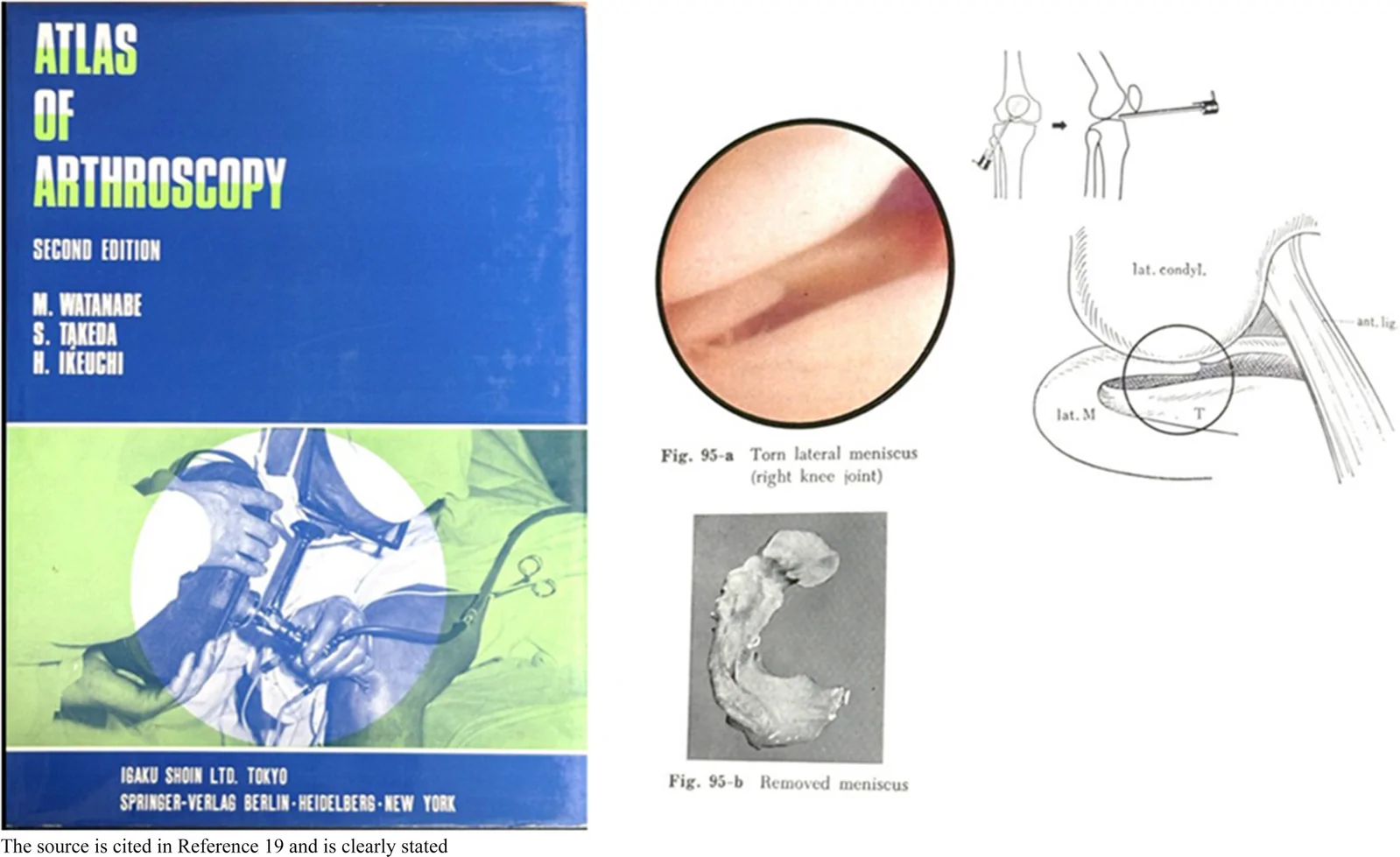
Atlas of Arthroscopy, 2nd edition in 1969 [21]

Subsequent development of the Watanabe arthroscope continued, leading to the introduction of the No. 24 arthroscope, a 1.7-mm self-focusing scope. Although its field of view was somewhat narrow, it provided high brightness, a deep depth of field, and clearer images than fiberoptic systems. With the introduction of the No. 24 scope, arthroscopic visualization became possible in nearly all joints of the body [17]. Development reportedly progressed further to the No. 31 arthroscope, a 3.0-mm instrument designed specifically for shoulder joint visualization [17] (Figs. 5, 6 and 7).

## International spread of Watanabe’s arthroscope

In 1964, Robert W. Jackson, a Canadian orthopaedic surgeon, met Watanabe in Tokyo during a traveling fellowship. He became the first international surgeon to learn arthroscopic techniques directly from Watanabe. Jackson subsequently purchased a Model-21 arthroscope and, immediately after returning to Canada, began performing arthroscopic procedures, completing approximately 70 cases by 1966 [22, 23]. In 1972, Jackson published a detailed report describing arthroscopic findings in 200 cases [24]. Jackson also introduced the anterolateral portal as a diagnostic entry point for visualization of the medial compartment, where the most frequently observed pathology was located [23].

S. Ward Cascells, the first editor of *Arthroscopy*, was the first surgeon in the United States to use Watanabe’s Model-21 arthroscope. He published his findings in 1971 under the title “Arthroscopy of the Knee Joint: A Review of 150 Cases,” which preceded Jackson’s reported by 1 year [12, 25, 26].

Richard L. O’Connor visited Watanabe in 1971 and learned the fundamental principles and techniques of knee arthroscopy. He subsequently developed a strong interest in operative arthroscopy and contributed to the development of arthroscopy training courses. He published his work under the title “Arthroscopy in the Diagnosis and Treatment of Acute Ligament Injuries of the Knee.” O’Connor is widely recognized for performing the first arthroscopic meniscectomy in North America in 1974 [27].

John B. McGinty and colleagues introduced the arthroscopic television system, which allowed multiple surgeons to observe surgical procedures simultaneously on a monitor. This innovation significantly enhanced surgical training and education and further improved the dissemination of arthroscopic techniques [12].

## Development of knee arthroscopy

As arthroscopy became more widely adopted, surgical techniques advanced rapidly, and both surgical indications and operative procedures evolved. In the early phase of development, the arthroscope was primarily used for diagnostic evaluation of the knee joint [28]. It was later extended to therapeutic applications.

From the late 1970 s through the 1980 s, rapid advances in arthroscopic techniques led to the widespread use of arthroscopic partial meniscectomy for meniscal tears. Because it was less invasive than open surgery and enabled faster recovery, arthroscopic partial meniscectomy became a standard treatment option. During the 1970 s, favourable outcomes were reported in relatively large clinical series [24], and the procedure became one of the most frequently performed orthopaedic operations. Although early arthroscopic interventions focused on meniscal resection, they later progressed toward meniscal preservation or, in other words, meniscal repair [29]. Notably, arthroscopic meniscal suture repair was first reported by Hiroshi Ikeuchi in 1969 [17, 30].

The world’s first ACL reconstruction was described by Hey Groves in 1917 as an open surgical procedure [31]. In the 1980 s, arthroscopy began to be used for ACL evaluation and reconstruction, marking a major advancement in ligament surgery [32–34].

In North America, arthroscopy continued to evolve and became increasingly associated with sports medicine. Joan Benoit drew widespread attention when she completed first in the Olympic trials only 17 days after undergoing arthroscopic knee surgery, subsequently qualifying for the 1984 Olympics, and winning the women’s Marathon at the Los Angeles Olympics three months later. Similarly, Mary Lou Retton underwent knee arthroscopy five weeks before the 1984 Olympics and went on to win the gold medal in the women’s individual all-around gymnastics competition. These high-profile cases strengthened the association between arthroscopy and athletic recovery, further accelerating its development and integration into sports medicine.

## Application of arthroscopy to joints other than the knee

In 1931, M. Burman conducted cadaveric studies of arthroscopic visualization of the shoulder, elbow, wrist, hip, and ankle joints; however, these techniques did not reach clinical application at that time [35]. In 1939, Kenji Takagi reported the clinical application of arthroscopy to the hip, shoulder, and ankle joints [36].

From the 1970 s onward, arthroscopy expanded beyond the knee and began to be applied clinically to multiple joints, including the shoulder [37]. In 1977, James M. Glick reported the use of hip arthroscopy [38]. Subsequently, arthroscopic techniques were extended to smaller joints, including the ankle [39, 40], wrist [41], and elbow [42].

In the 1990 s, the development of microendoscopic discectomy using spinal endoscopy marked the introduction of endoscopic techniques into spinal surgery [43, 44]. Today, arthroscopic and endoscopic techniques are indispensable in orthopaedic surgery, enabling a wide range of minimally invasive procedures across multiple joints and anatomical regions.

## Data Availability

No datasets were generated or analysed during the current study.
